# Radiotherapy-induced enrichment of EGF-modified doxorubicin nanoparticles enhances the therapeutic outcome of lung cancer

**DOI:** 10.1080/10717544.2022.2036871

**Published:** 2022-02-14

**Authors:** Jing Wang, Yan Zhang, GuangPeng Zhang, Li Xiang, HaoWen Pang, Kang Xiong, Yun Lu, JianMei Li, Jie Dai, Sheng Lin, ShaoZhi Fu

**Affiliations:** a Department of Oncology, the Affiliated Hospital of Southwest Medical University, Luzhou, China; b Department of Oncology, The Affiliated TCM Hospital of Southwest Medical University, Luzhou, China

**Keywords:** Doxorubicin, nanoparticles, EGF, lung cancer, radiotherapy

## Abstract

Chemotherapy is the primary treatment for advanced non-small-cell lung cancer (NSCLC). However, related dose-dependent toxicity limits its clinical use. Therefore, it is necessary to explore new strategies for improving the clinical outcomes while reducing the side effects of chemotherapy in the treatment of NSCLC. In this study, we designed and synthesized epidermal growth factor (EGF)-modified doxorubicin nanoparticles (EGF@DOX-NPs) that selectively targets the epidermal growth factor receptor (EGFR) overexpressed in lung tumor cells. When administered in combination with low-dose X-ray radiotherapy (RT), the NPs preferentially accumulated at the tumor site due to radiation-induced outburst of the local intra-tumoral blood vessels. Compared with DOX alone, EGF@DOX-NPs significantly decreased the viability and migration and enhanced the apoptosis rates of tumor cells *in vitro*. Also, the EGF@DOX-NPs significantly inhibited tumor growth *in vivo*, increasing the survival of the tumor-bearing mice without apparent systemic toxic effects through RT-induced aggregation. The tumor cell proliferation was greatly inhibited in the RT + EGF@DOX-NPs group. Contrarily, the apoptosis of tumor cells was significantly higher in this group. These results confirm the promising clinical application of radiotherapy in combination with EGF@DOX-NPs for lung cancer treatment.

## 1. Introduction

Lung cancer is the leading cause of cancer-related deaths in China, and non-small cell lung cancer (NSCLC) accounts for ∼85% of all cases (Arbour & Riely, 2019; Tang et al., 2021). Chemotherapy remains one of the primary treatments for advanced NSCLC (Engelberg et al., 2019). However, due to the nonspecific targeting of the conventional chemotherapy drugs and the complexity of the tumor microenvironment (TME), drug accumulation in tumor tissues is suboptimal, and the therapeutic effects are not satisfactory (Matarrese et al., 2021; Yu et al., 2021). Although increasing the drug dose would elevate its intratumoral concentration to therapeutically optimal levels, it will also increase dose-dependent toxicity (Budker et al., 2014). In addition, targeted delivery of chemotherapy drugs to tumor tissue can improve therapeutic efficacy and limit side effects. However, the characteristic features of the TME frequently impede drug penetration (Mohanty et al., 2020). Therefore, novel strategies to improve drug concentration at the target site are urgently needed.

Increasing evidence shows that nanoparticle (NP)-based drug delivery systems have unique advantages such as better water solubility, stability, controlled release, and selective targeting, all of which increases drug accumulation in tumors (Chen et al., 2021; Li et al., 2021 ; Mohtar et al., 2021; Qin et al., 2021). Some nano-formulations such as doxorubicin (DOX) liposomes and albumin-embedded paclitaxel nanoparticles have already entered clinical practice (Overchuk & Zheng, 2018; Li & Zhang, 2019). These NPs passively accumulate in the tumor tissues due to the enhanced permeability and retention (EPR) effect (Rosenblum et al., 2018; Shang et al., 2021; Zhang et al., 2021). In addition, NPs functionalized with affinity ligands or antibodies can actively target tumor cells (Li et al., 2018; Biffi et al., 2019). Studies show that compared to normal cells, epidermal growth factor receptor (EGFR) is overexpressed on the surface of NSCLC cells and, as such, is a suitable marker for active targeted therapy (Zhao et al., 2018). Hence, surface modification of NPs with EGF can significantly improve drug uptake by tumor cells and increase its concentration in tumor tissues through ligand-receptor-specific binding.

However, the high interstitial pressure, low microvascular permeability, hypoxia, and acidosis in the TME limit the concentration of drugs to sub-therapeutic levels (Kim et al., 2017; Gallo et al., 2021). In addition, the abnormal and aberrant vasculature in solid tumors significantly affects chemoradiotherapy outcomes (Fokas et al., 2012; Viallard & Larrivée, 2017). Studies show that a single dose of 5 Gy radiotherapy can induce transient, dynamic, and local ‘burst’ of tumor microvessels, thereby increasing vascular permeability and increasing drug accumulation in tumors (Miller et al., 2017). In addition, the disruption of tumor microvessels also releases the tumor-associated macrophages (TAMs), which then infiltrate into the tumor tissue and further enhance nano-drug accumulation (Miller et al., 2015). Thus, radiation-induced disruption of the tumor microvessels is also a promising strategy for improving the accumulation of drug-loaded NPs in tumor tissues.

**Scheme 1.**
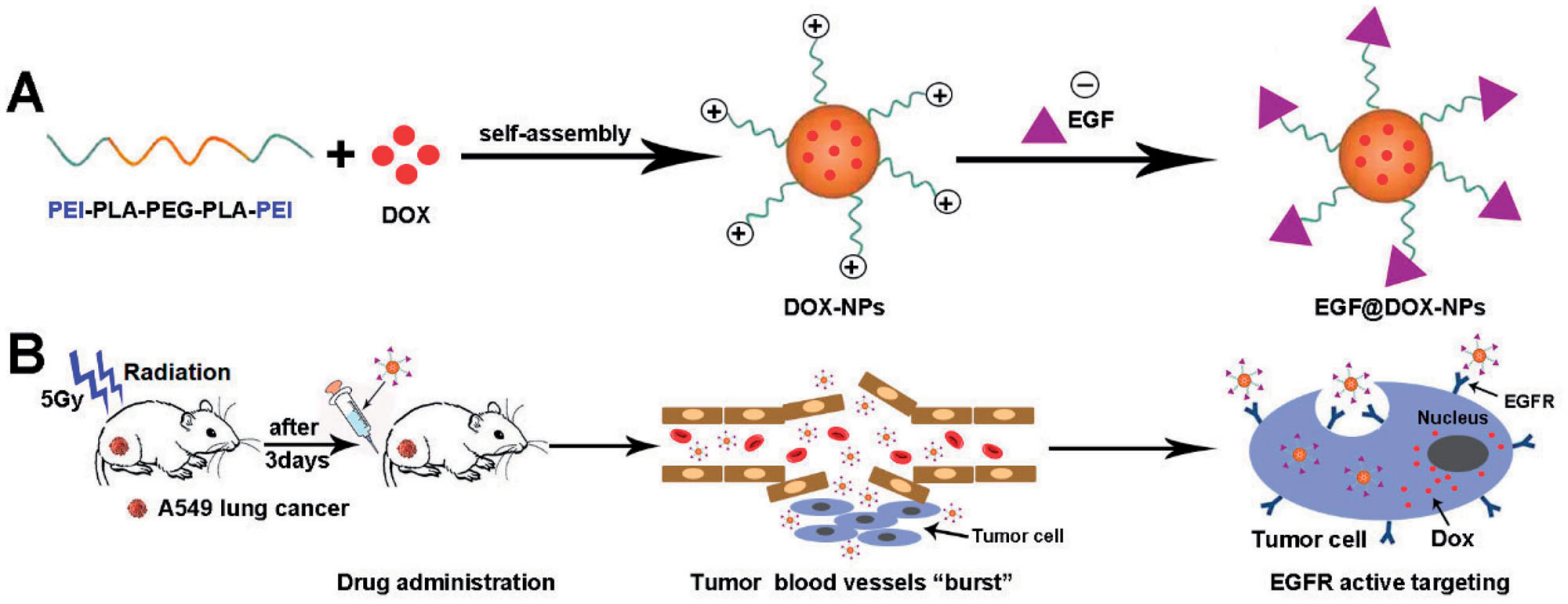
Schematic diagram of the preparation of NPs and the principle of radiation-induced drug aggregation.

We synthesized DOX-loaded NPs using polyethylenimine (PEI) coated on biocompatible polylactic acid–polyethylene glycol–polylactic acid copolymer (PLA-PEG-PLA) (Zhang et al., 2010; Massadeh et al., 2021). Surface charge neutralization was performed using EGF. The EGF@DOX-NPs, in combination with radiotherapy, exhibited selective cytotoxicity to lung tumor cells and significantly inhibited the growth of lung tumor xenografts *in vivo* (Scheme 1).

## 2. Materials and methods

### 2.1. Synthesis and characterization of PEI-PLA-PEG-PLA-PEI copolymer

The triblock PLA-PEG-PLA copolymer (PELA, Mw = 8000) was synthesized through ring-opening polymerization. Briefly, 1.25 mM PEG (Mw = 4000) and 2.5 mM d,l-LA were mixed in a three-necked flask containing Sn(Oct)_2_ (0.5% of the total weight of all reactants). The reaction was carried out for 6 h at 130 °C in the presence of N_2_. The product was repeatedly precipitated with pre-cooled petroleum ether, and dried in a vacuum oven. To synthesize the copolymer PEI-PLA-PEG-PLA-PEI (PELI), 1 mM PELA was first dissolved in 40 mL of dichloromethane and reacted with 2 mM triethylamine and 4 mM acryloyl chloride for 6 h at 40 °C under N_2_. The product was precipitated with pre-cooled petroleum ether, vacuum-dried, and 1 mM HOOC-PELA-COOH copolymer was dissolved in 20 mL of chloroform. Finally, 2 mM PEI solution in methanol was slowly added, and the reaction was performed at 50 °C for 24 h under N_2_. The product was precipitated and dried (yield ∼60%). The molecular structures of PELA and PELI were determined by proton nuclear magnetic resonance (^1^H-NMR) and Fourier transformation infrared (FTIR) spectroscopy.

### 2.2. Preparation and characterization of DOX-NPs and EGF@DOX-NPs

DOX.HCl (10 mg or 15 mg) and PELI (90 mg or 85 mg) were dissolved in 5 mL of methanol and mixed uniformly. The organic solvent was utterly removed at 37 °C, and the precipitate was dissolved in 10 mL of deionized water at 37 °C. After removing the nonencapsulated drug by ultrafiltration (molecular weight cutoff 3 kDa, 4000 rpm for 15 min), the solution was passed through a 220-nm filter and then lyophilized. The stability of the DOX-NPs was evaluated by continuously measuring the particle size every 6 h for 30 h. The blank PELA-NPs and PELI-NPs were prepared in the same manner without drug encapsulation. EGF@DOX-NPs were synthesized by sonicating the DOX-NPs (1 mg DOX) for 5–10 min with 100 µL aqueous EGF (0.1 mg/mL). The unreacted protein was removed by centrifugation to obtain pure EGF@DOX-NPs. The particle size and zeta potential of the different NPs were measured using dynamic light scattering (DLS, NanoBrook 90 Plus Zeta, Brookhaven, NY), whereas their morphology was observed using a transmission electron microscope (TEM, ZEISS Libra 200 FE, Germany). The drug loading (DL) capacity and encapsulation efficiency (EE) of the DOX-NPs were determined using an ultraviolet spectrophotometer (UV-5800PC, Shanghai, China) at 480 nm according to the following formulae:

$$\mathrm{DL}\;=\;\frac{\mathrm{DOX}(\mathrm{total})\;-\;\mathrm{DOX}(\mathrm{unencapsulated})}{\mathrm{DOX}(\mathrm{total})\;+\;\mathrm{PELI}}\;\times \;100\%$$

$$\mathrm{EE}\;=\;\frac{\mathrm{DOX}(\mathrm{total})\;-\;\mathrm{DOX}(\mathrm{unencapsulated})}{\mathrm{DOX}(\mathrm{total})}\;\times \;100\%$$

### 2.3. *In vitro* release behavior

The different DOX formulations (free DOX, DOX-NPs, and EGF@DOX-NPs) containing the equivalent amount of the drug were dissolved in deionized water and dialyzed (molecular weight cutoff of the dialysis bag – 3.5 kDa) against 40 mL phosphate buffer saline (PBS) (pH = 7.4) and 1% Tween 80 (vol/vol) at 37 °C with constant shaking at 100 rpm. At regular intervals, 3 mL of the dialysis buffer was removed and replenished with an equal amount of fresh buffer. The amount of the released drug was measured using an ultraviolet spectrophotometer.

### 2.4. *In vitro* cytotoxicity, cell apoptosis, and intracellular uptake of NPs

A549 cells were seeded into a 96-well plates at the density of 1.8 × 10^4^ cells/well, and treated with different drug concentrations for 48 h. Freshly prepared 3-(4,5-Dimethylthiazol-2-yl)-2,5-diphenyltetrazolium bromide (MTT) solution (5 mg/mL, 20 µL) was added to each well before 4-h incubation. After dissolving the formazan crystals with dimethyl sulfoxide (DMSO) (150 µL per well), the absorbance at 490 nm was measured using an iMark microplate reader (Bio-RAD, USA). To evaluate apoptosis, the A549 cells were seeded in 6-well plates at the density of 1 × 10^6^ cells/well and incubated with the different formulations (10 µg/mL DOX) for 24 h. The cells were harvested, washed, and stained with 5 µL Annexin V-mCherry and 1 µL SYTOX Green Staining Solution in 200 µL buffer for 15 min in the dark. The rate of apoptosis cells was obtained using flow cytometry (Beckman Coulter, DxFlex, USA). To measure the rate of drug uptake, A549 (A549 human non-small lung cancer cell), AML-12 (alpha mouse liver 12), and BEAS-2B (bronchial epithelium transformed with Ad12-SV40 2B) cells were seeded into a 6-well plates at the density of 2 × 10^5^ cells/well, and incubated in free DOX, DOX-NPs, EGF@DOX-NPs or EGF + EGF@DOX-NPs (equivalent to 2.5 µg/mL DOX) for 2 h. The cells were then observed using an inverted fluorescence microscope (OLYMPUS, IX73, Japan) and intracellular uptake was quantified using flow cytometry. The relative expression levels of EGFR in A549, AML-12, and BEAS-2B cells were assessed using Western blot analysis.

### 2.5. Wound healing assay

A549 cells were seeded in 6-well plates at the density of 1 × 10^6^ cells/well. Upon reaching (90%–100%) confluence, the monolayer was scratched longitudinally with a sterile pipette tip, and the medium was discarded. Fresh medium containing the different formulations (1 µg/mL DOX) was then added. The wound area was observed at 0, 6, 12, and 24 h under a microscope (OLYMPUS, CKX53, Japan).

### 2.6. Hemolysis assay

Blood samples were incubated at 37 °C for 4 h with different concentrations of the drugs. PBS and deionized water were used as the negative control and positive control, respectively. Hemolysis was assessed through visual examination, whereas the cellular morphology was observed using a microscope (OLYMPUS, CKX53, Japan). The amount of hemoglobin released from the lysed RBCs was measured using an ultraviolet spectrophotometer (UV-5800PC, Shanghai, China) at 415 nm. The hemolysis rate was then calculated according to the following formula:

$$\text{Hemolysis rate }=\;\frac{\mathrm{Value}(\mathrm{Drug})\;–\;\mathrm{Value}(\mathrm{Negative})}{\mathrm{Value}\;(\mathrm{Positive})}\;\times \;100\%$$

### 2.7. *In vivo* evaluation of the antitumor effect

Lung cancer xenografts were established by inoculating the right thigh of BALB/c-nu with 2 × 10^6^ of A549 cells. After the tumor had grown to 50–100 mm^3^, the mice were randomly divided into seven treatment groups as follows: (1) control, (2) radiotherapy (RT), (3) free DOX, (4) EGF@DOX-NPs, (5) RT + free DOX, (6) RT + DOX-NPs, and (7) RT + EGF@DOX-NPs groups. The mice were irradiated once with a single 5 Gy and injected intravenously with the different formulations (equivalent to 5 mg/kg DOX) after 3 days, three times once every 3 days. The body weight of the mice and tumor volume were measured every two days. The overall survival period of the mice was also recorded. At the end of the treatment regimen, the mice were euthanized before extracting major organs (heart, liver, spleen, lung, and kidney) and the tumors. The drug content in these samples was determined using an LS55 luminescence spectrometer (Perkin-Elmer, USA) at the emission wavelength of 584 nm.

Glucose metabolism in the tumors was analyzed using micro-positron emission tomography / computed tomography (PET/CT) imaging. Briefly, after drug administration, the mice were starved for 8–12 h before being injected with 150–200 µCi [(18)F]-fluorodeoxy-D-glucose (^18^F-FDG）. After 30 min, the mice were anesthetized and scanned by the PET/CT imaging system using the following parameters: 80 kV, 500 mA, and slice thickness 1.5 mm. The maximum standardized uptake value (SUV_max_) of tumor regions was calculated based on the region of interest (ROI) of tumor tissues.

### 2.8. Histopathological and immunohistochemical analysis

The harvested organs were fixed in formalin, dehydrated with 70% alcohol, embedded in paraffin, sectioned, and stained with hematoxylin and eosin (H&E) as per standard protocols. The stained sections were observed under a light microscope (OLYMPUS, IX73, Japan) to evaluate histological changes. TdT-mediated dUTP nick end labeling (TUNEL) staining and immunohistochemical staining for Ki-67, CD34, and F4/80 were performed following the manufacturer’s protocols.

### 2.9. *In vivo* safety assessment

The biosafety of the different DOX formulations was evaluated based on hematological indices. The drugs were injected intravenously three times, once every 3 days. The animals were euthanized 24 h after the last treatment before extracting blood for cell counts and biochemical analysis.

### 2.10. Statistical analysis

Data were analyzed using GraphPad Prism software, version 6.07 (GraphPad Software, Inc) and expressed as mean ± standard deviation (SD). Differences between groups were analyzed using Student’s *t* test and one-way analysis of variance (ANOVA). *p* < .05 was considered statistically significant.

## 3. Results

### 3.1. Characterization of PELA and PELI co-polymers

The procedure for the synthesis of PELA and PELI co-polymers is outlined in Supplementary Figure S1(A). Their chemical structures were assessed using ^1^H-NMR and FTIR. The proton peaks of methyl (CH_3_) and methane (CH) in PLA were around 1.30 ppm and 4.75 ppm, respectively, whereas the methylene (CH_2_) protons in PEG were 3.65 ppm. After PEI grafting, the peaks of the amino (NH_2_) protons were around 2.51 ppm, 2.71 ppm, and 2.88 ppm (Supplementary Figure S1(B)). PELA-FITR spectra (Supplementary Figure S1(C)) revealed that hydroxyl (O-H), carboxyl ester (C = O), and diethyl ether (C-O-C) groups generated sharp peaks of 3440 nm^−1^, 1750 nm^−1^, and 1110 nm^−1^, respectively. After PEI modification, the peak value of 3440 nm^−1^ became weaker and a new amino (N-H) group peak appeared at 3380 nm^−1^. In addition, the strength of the peak at 1750 nm^−1^ weakened and shifted from 1650 nm^−1^ to 1200 nm^−1^, indicating the formation of new amide bonds. Thus, the PELI copolymer was successfully synthesized.

### 3.2. Characterization of NPs

**Figure 1.**
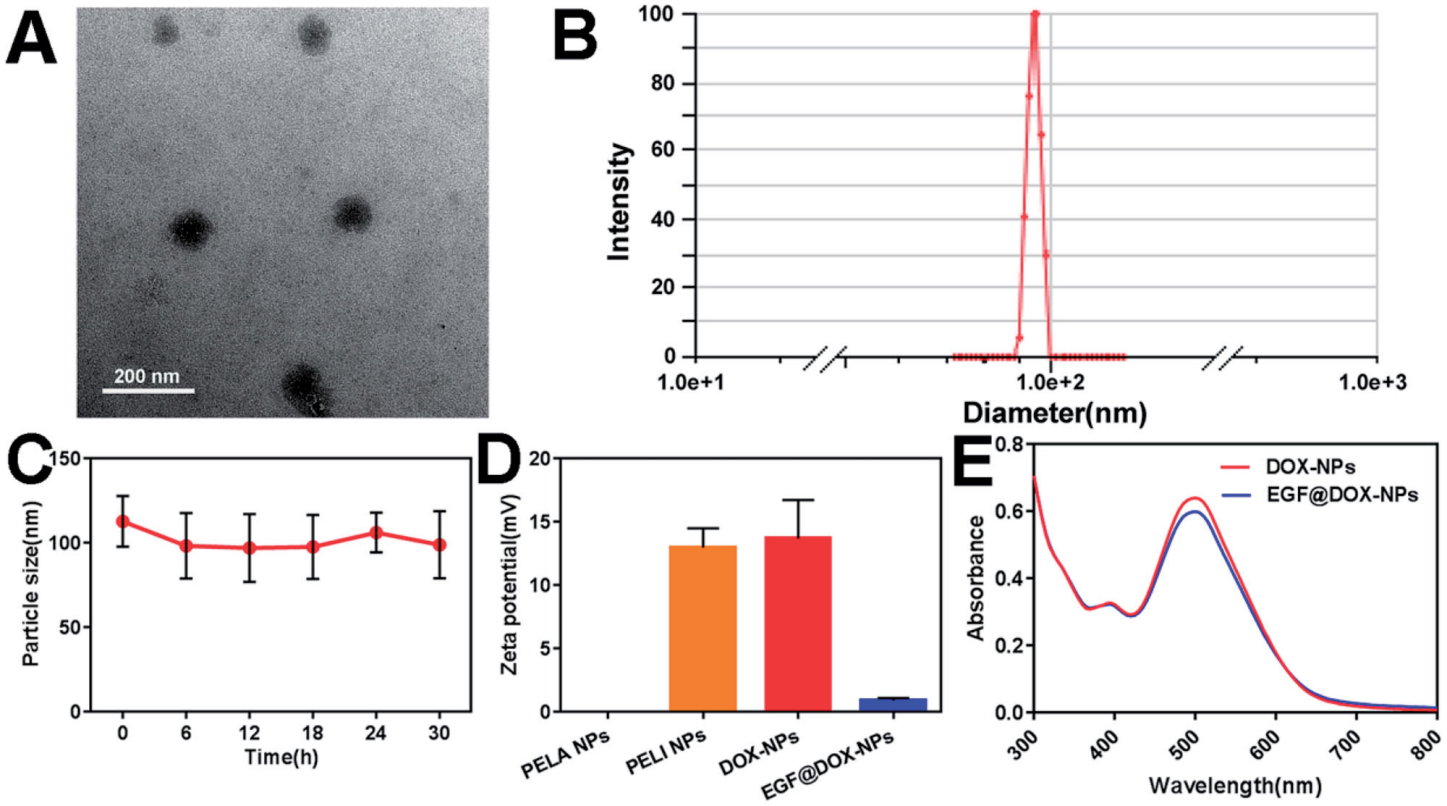
In vitro characterization of DOX NPs. (A) TEM image of DOX-NPs. (Scale bar: 200 nm). (B) Size distribution of DOX-NPs determined by DLS. (C) The particle size change of DOX-NPs in PBS. (D) Zeta potential of the blank PELI NPs and DOX NPs. (E) UV–vis absorbance curves of DOX-NPs and EGF@DOX-NPs. (Mean ± SD; *n* = 3).

As shown in Supplementary Table S1, the actual DL and EE of DOX-NPs were both greater than 90%. The TEM images (Figure 1(A)) further indicated even dispersion of spherical NPs of diameter ∼100 nm, consistent with DLS results (Figure 1(B)). Furthermore, the shape and size of the DOX-NPs were unaltered after 30 h of storage (Figure 1C), demonstrating their high stability. As shown in Figure 1(D), compared with the electroneutral PELA NPs, the zeta potential of PELI NPs significantly increased to +12.98 ± 1.53 mV, attributed to PEI grafting. After DOX loading, the potential slightly increased to +13.70 ± 3.05 mV, whereas that of EGF@DOX-NPs decreased to +0.90 ± 0.2 mV due to the negatively charged EGF. As shown in Figure 1(E), the absorbance intensity of EGF@DOX-NPs slightly decreased after EGF coating compared to that of DOX-NPs. These results indicated the successful preparation of EGF@ DOX-NPs through charge neutralization.

### 3.3. *In vitro* drug release and cytotoxicity

**Figure 2.**
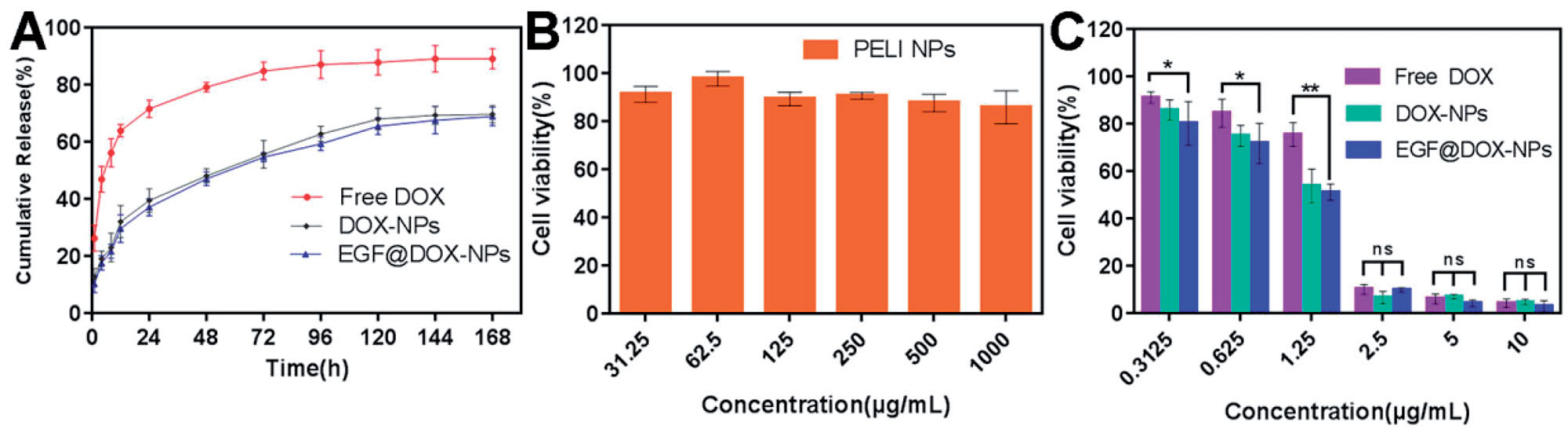
*In vitro* drug release and cytotoxicity. (A) The cumulative release of DOX from different DOX formulations within 7 days. (B) The viability of A549 cells treated with blank PELI NPs for 48 h. (C) The viability of A549 cells treated with free DOX, DOX-NPs, and EGF@DOX-NPs for 48 h. (Mean ± SD; *n* = 3). ns: no significance, *p < .05, ** *p* < .01.

As shown in Figure 2(A), compared with the encapsulated drug, the release of free DOX was significantly fast, and the cumulative release amount exceeded 70% within 24 h and peaked after 72 h. The cumulative release of DOX from the NPs was less than 40% within 24 h, and the drug was released steadily in a time-dependent manner. A similar pattern was observed with the EGF@DOX-NPs, indicating that EGF modification had no significant effect on drug release.

The cytotoxicity of blank PELI NPs and different DOX formulations were evaluated on A549 cells. The blank PELI NPs had no apparent toxic effect. As shown in Figure 2(B), the viability of the cells treated with a high concentration (1000 µg/mL) of the NPs was close to 85%, indicating that the PELI copolymer was biocompatible. On the other hand, the DOX formulations displayed concentration-dependent cytotoxicity (Figure 2(C)), with 2.5 µg/mL of the formulation inhibiting the proliferation of 90% of the cells. The cytotoxic effects of the three-drug formulations were generally similar, although low concentrations of EGF@DOX-NPs were significantly more toxic than those of the free DOX (*p* < .05). These results indicate that DOX release from EGF@DOX-NPs was continuous and exerted a stronger antitumor effect.

### 3.4. Cell apoptosis, intracellular uptake, and wound healing

**Figure 3.**
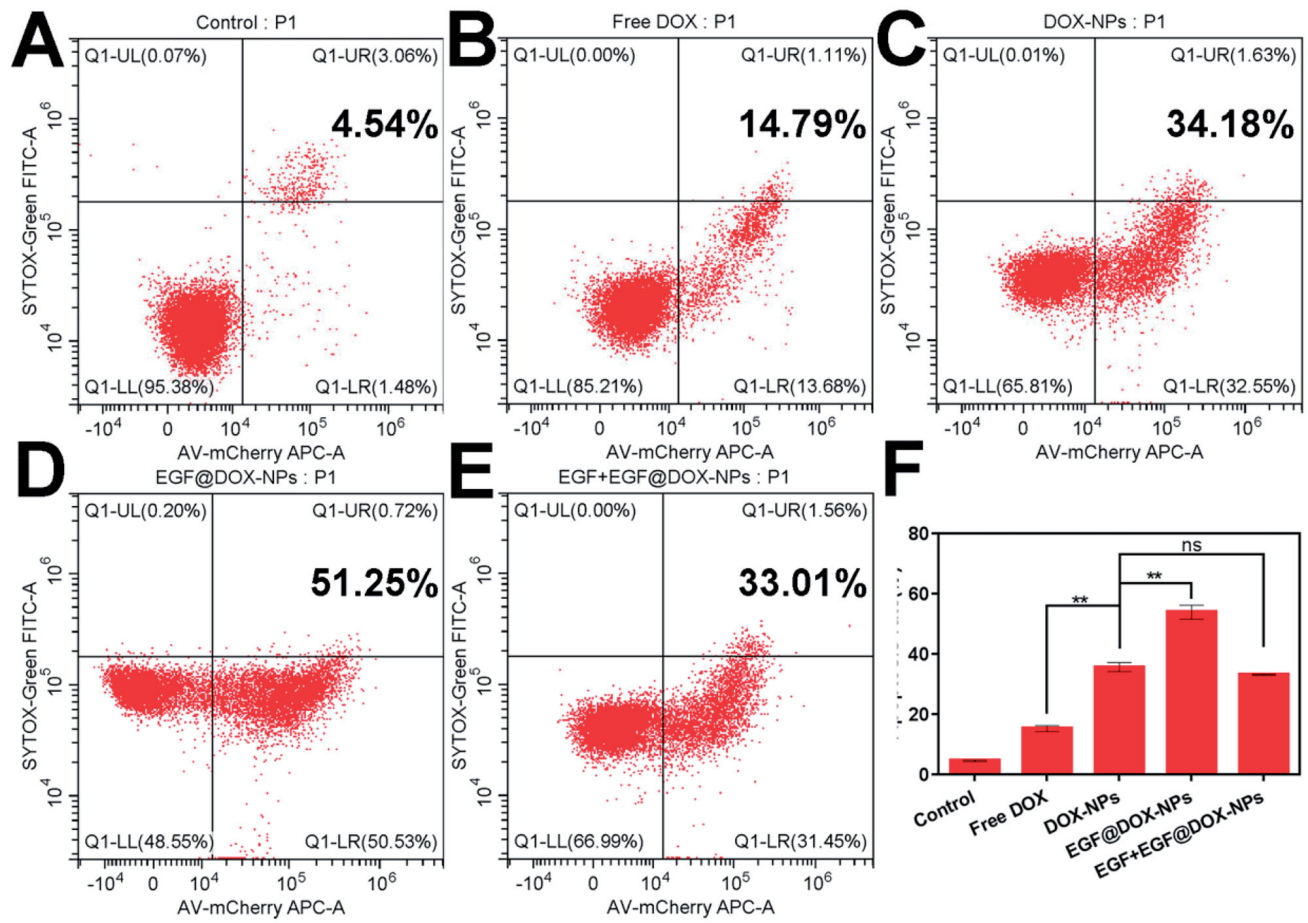
*In vitro* apoptosis. (A–E) Typical flow cytometry profiles showing apoptotic cells (DOX concentration: 10 µg/mL). (F) Apoptosis rate of A549 cells treated with different drugs for 24 h. (Mean ± SD; *n* = 3). ns: no significance, ** *p* < .01.

Compared to free DOX (15.19% ± 0.95%), DOX-NPs significantly increased the apoptosis of A549 cells (35.6% ± 1.53%). The percentage of apoptotic cells exceeded 50% when treated with EGF@DOX-NPs (*p* < .01 compared to all groups), although pretreatment with EGF led to a significant decrease in apoptosis rates (33.10% ± 0.21%) similar to that of DOX-NPs group (Figure 3).

**Figure 4.**
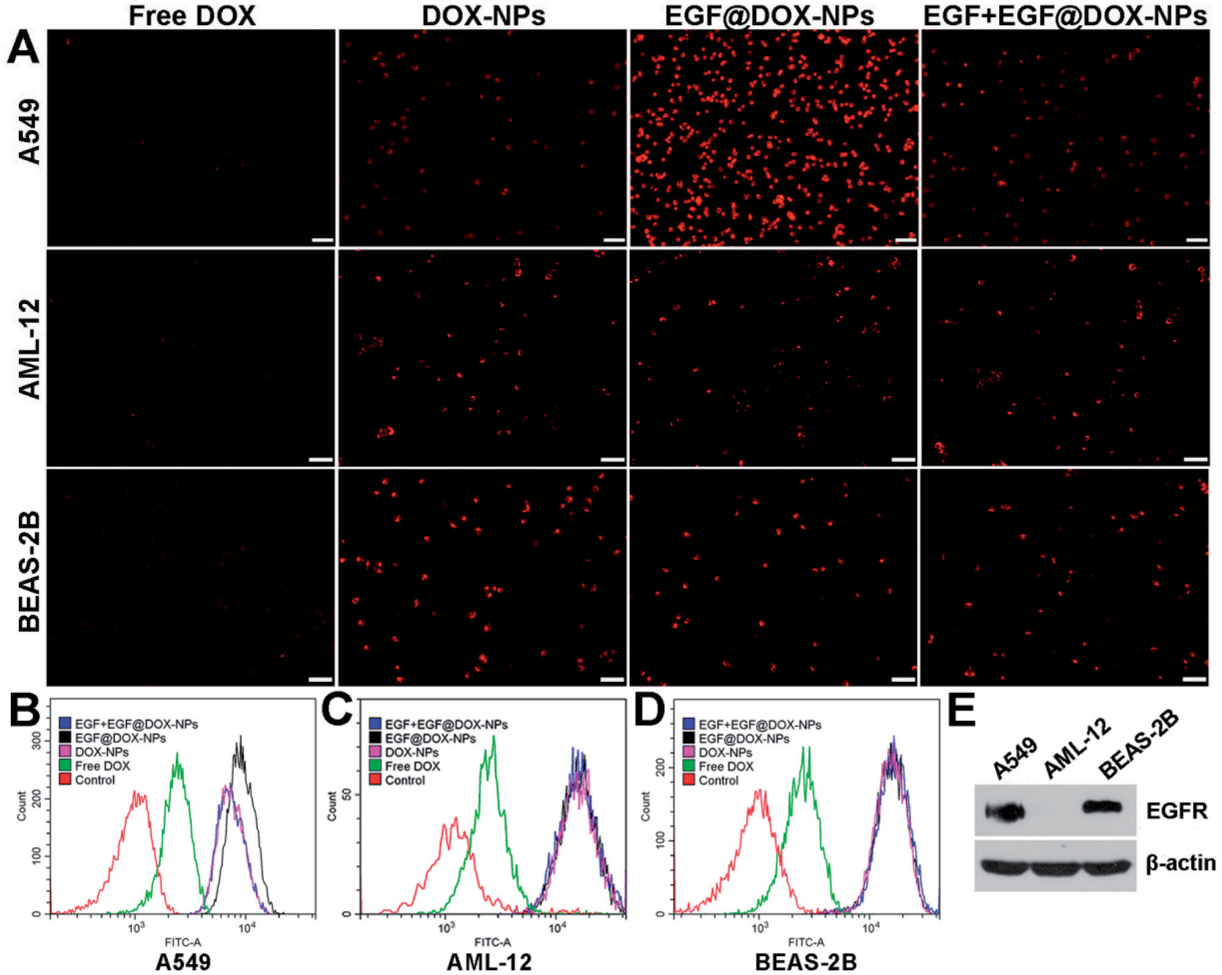
*In vitro* cell uptake. (A) Fluorescence microscopy images showing the uptake of DOX NPs into the EGFR^high^ A549 cells, and the EGFR^low^ AML-12 and BEAS-2B cells. (DOX concentration: 2.5 µg/mL, EGF pretreatment for 4 h in the EGF + EGF@DOX-NPs group; Scale bar: 100 µm). (B–D) DOX content in the A549, AML-12, and BEAS-2B cells was determined by flow cytometry. (E) EGFR protein expression in the A549, AML-12, and BEAS-2B cells was assessed by Western blot analysis. β-Actin expression served as a loading control.

Furthermore, the uptake of the NPs in the A549, AML-12, and BEAS-2B cells was significantly higher than that of free DOX (Figure 4(A)). The fluorescence intensity of A549 cells incubated with EGF@DOX-NPs was the highest, attributed to the targeted uptake of the NPs given the increased surface expression of EGFR on A549 cells compared to AML-12 and BEAS-2B cells. Meanwhile, EGF pretreatment for 4 h significantly inhibited the EGF@DOX-NPs uptake. In contrast, EGF pretreatment had no effect on the uptake of EGF@DOX-NPs by AML-12 and BEAS-2B cells (Figure 4(B–D)). Western blot analysis showed that EGFR expression was relatively high in A549 cells compared with AML-12 and BEAS-2B cells (Figure 4(E)). Thus, EGF@DOX-NPs uptake in A549 cells occurs via EGFR-mediated endocytosis.

**Figure 5.**
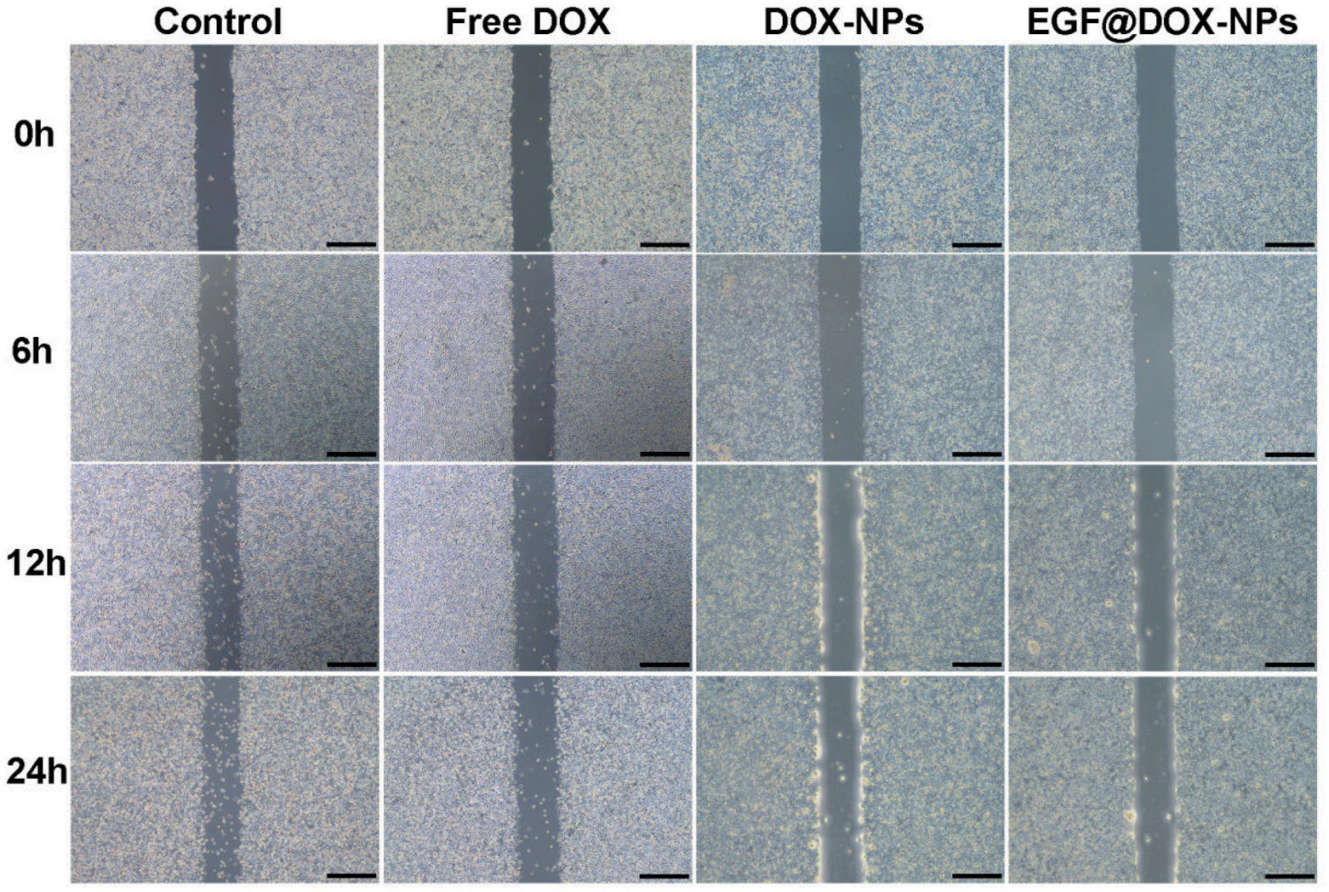
Typical images of wound healing of A549 monolayer after treatment with different drugs for 0, 6, 12, and 24 h (DOX concentration: 1 µg/mL; Scale bar: 500 µm).

The inhibitory effect of different DOX formulations on cell migration was analyzed using wound-healing assays. As shown in Figure 5, the untreated tumor cells gradually migrated to the wound region in a time-dependent manner. In contrast, treatment with free DOX, DOX-NPs, or EGF@DOX-NPs inhibited this phenomenon *in vitro*. Nevertheless, while cell migration was still visible in the free DOX-treated group, DOX-NPs and EGF@DOX-NPs treatment almost completely inhibited this phenomenon. Thus, nanosized DOX can inhibit the migration of tumor cells more effectively than free DOX.

### 3.5. *In vitro* hemolysis assay

**Figure 6.**
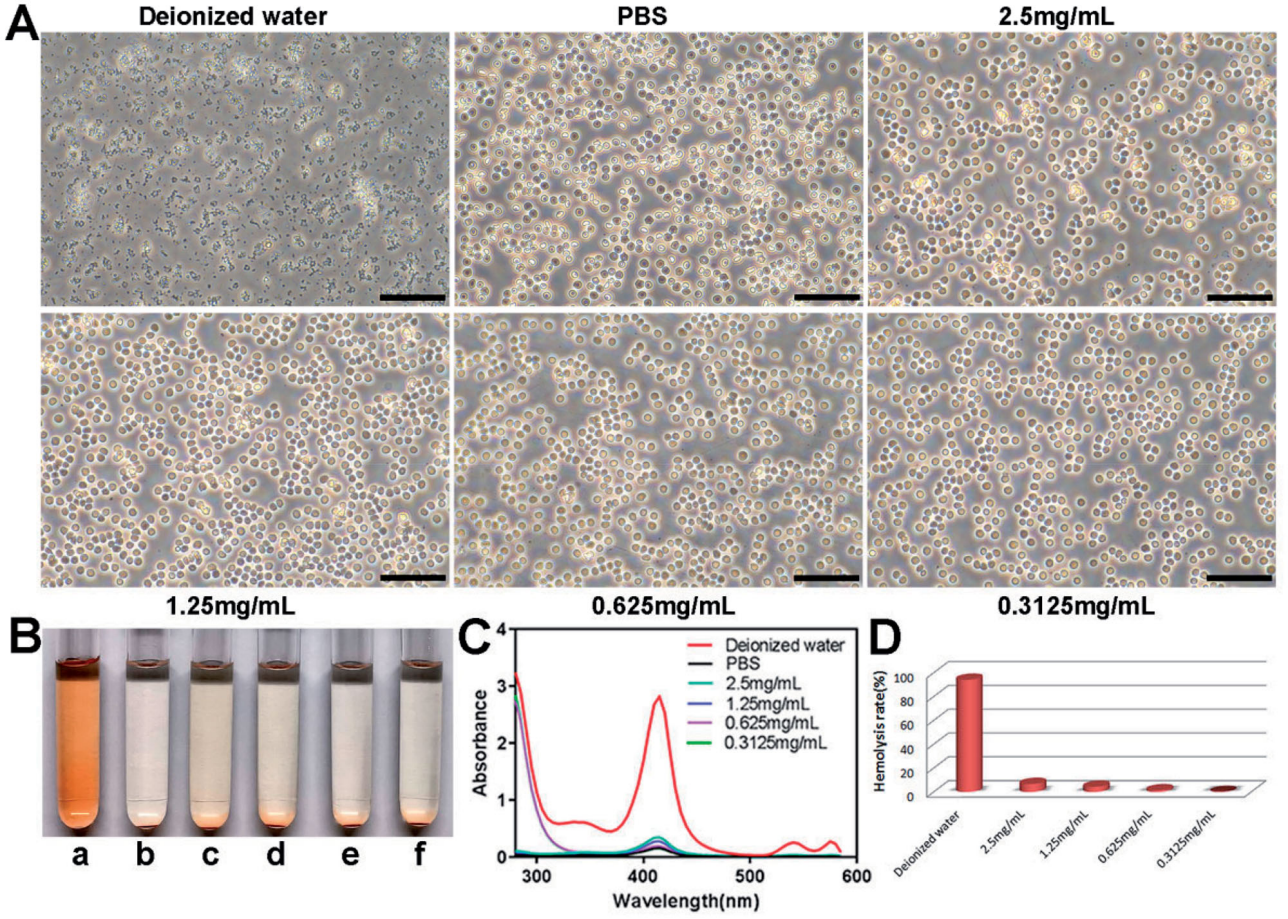
*In vitro* hemolysis analysis of the PELI NPs with different concentrations. (A) Morphological changes of erythrocytes in different groups after 4 h co-incubation (Scale bar: 50 µm). (B) The representative images of different groups (a: deionized water; b: PBS; c–f: the PELI NPs with concentration of 2.5 mg/mL,1.25 mg/mL, 0.625 mg/mL, 0.3125 mg/mL, respectively). (C) UV absorbance curves of hemoglobin in the different groups. (D) Hemolysis rate in each group.

The blood compatibility of the PELI NPs was evaluated using the hemolysis assay. As shown in Figure 6(A), the red blood cells were uniformly distributed and retained their shape in PBS, whereas numerous cellular fragments were observed in deionized water, indicating severe hemolysis. The blank PELI NPs had little effect on the morphology of red blood cells. Furthermore, the bright red color of deionized water indicated hemolysis, whereas the other solutions only had a pale tinge (Figure 6(B)). Consistently, ultraviolet absorbance at 415 nm was strongest for deionized water (Figure 6(C,D)) and very low even in the presence of 2.5 mg/mL PELI NPs, indicating good biocompatibility of the nanocarriers.

### 3.6. *In vivo* antitumor effect

**Figure 7.**
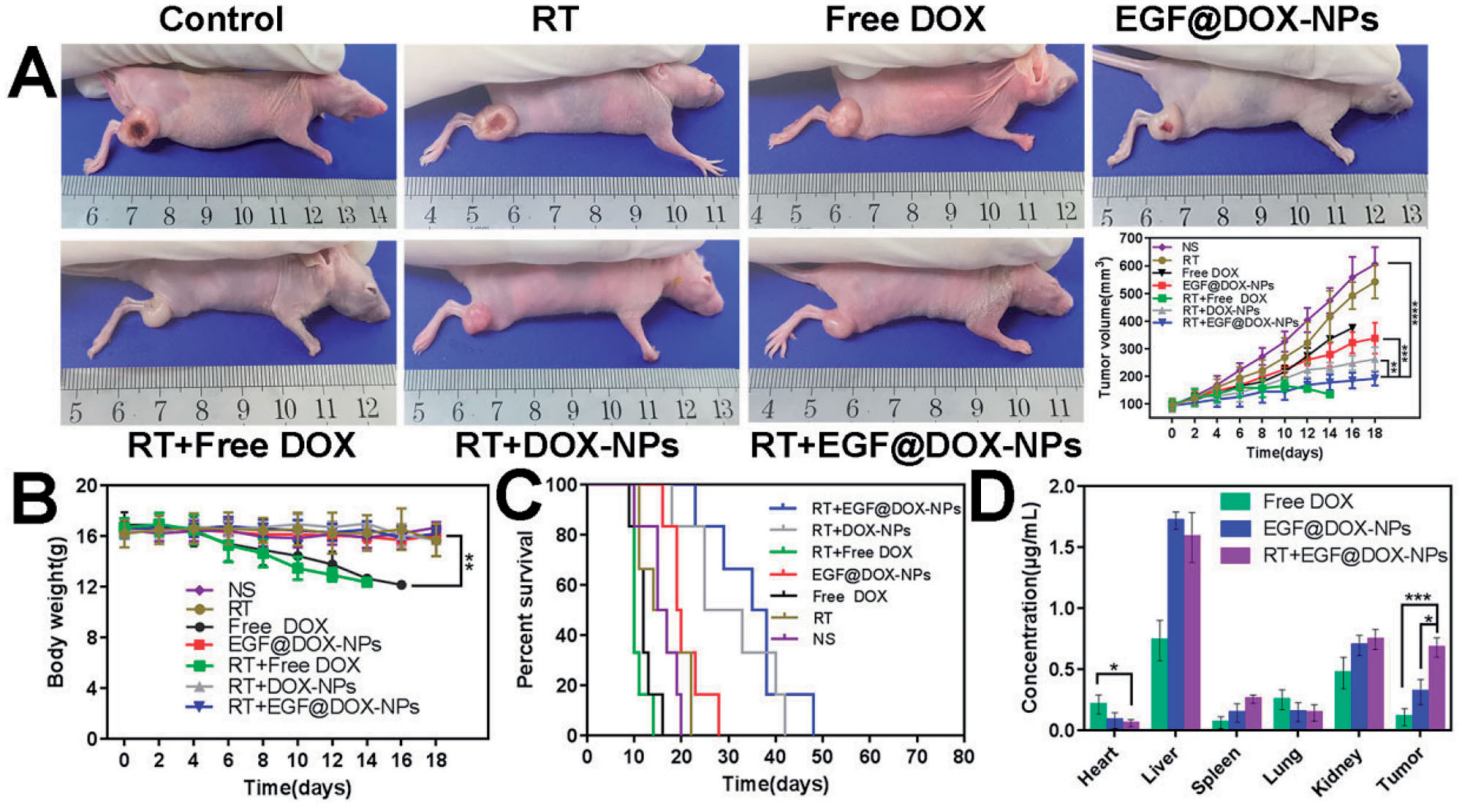
*In vivo* evaluation of antitumor effects and drug biodistribution. (A) Representative images of mice in each group and the change in tumor volume after different interventions. (B) The change in body weight of mice in the different groups. (C) Survival curves of mice in each group. (D) DOX distribution in the vital organs (including heart, liver, spleen, lung, and kidney) and tumors in the free DOX, EGF@DOX-NPs, and RT + EGF@DOX-NPs groups. ** *p* < .01, *** *p* < .001, **** *p* < .0001.

The *in vivo* antitumor effects of the NPs were evaluated using lung tumor xenografts in a mouse model. As shown in Supplementary Fig. S2(A,B), the tumors in the control mice were significantly larger compared to those in the treated groups. The combination of RT with free DOX or EGF@DOX-NPs exhibited the strongest inhibitory effect on tumor growth (Figure 7(A)), and the growth rate was lowest in the RT + EGF@DOX-NPs group (*p* < .01). However, the mice treated with free DOX with or without RT were emaciated and lost significant weight with time (Figure 7(B)), which can be attributed to the systemic side effects of DOX. Furthermore, compared with the other treatment groups and control (16 days), the overall survival of mice that received only DOX treatment (12.5 days) or RT + free DOX (10 days) was significantly shorter. Mice in the RT + EGF@DOX-NPs group displayed the longest median survival duration of 36.5 days, compared to 15.5, 19.5, and 29 days for mice in the RT, EGF@DOX-NPs, and RT + DOX-NPs groups, respectively (*p* < .05).

The antitumor efficacy is closely related to the drug content in tumor tissue (Figure 7(D)). Due to the first-pass elimination effect, DOX in the liver was relatively higher in all groups. However, encapsulation of DOX in nanoparticles and EGF modification significantly decreased the accumulation of the drug in the cardiac tissues and increased its content in the tumor tissues (0.11 ± 0.07 µg/mL of free DOX vs 0.32 ± 0.10 µg/mL of EGF@DOX-NPs). The highest intratumoral DOX levels were observed in the RT + EGF@DOX-NPs group at 0.68 ± 0.08 µg/mL (*p* < .05). Overall, EGF modification mediated selective accumulation of DOX-NPs in the tumor tissues, and RT further enhanced the drug content to therapeutically significant levels.

### 3.7. H&E and immunohistochemical analysis

Systemic toxicity was assessed using histopathological changes in tissues of major organs. As shown in Supplementary Figure S3, extensive hemorrhaging was observed in the myocardial tissues of mice treated with free DOX with or without RT, which can be attributed to DOX-mediated cardiotoxicity. In contrast, the arrangement of myocardial fibers in the other groups was generally normal. Except for the RT + DOX-NPs and RT + EGF@DOX-NPs groups, varying degree of liver metastasis was observed in the other groups, indicative of a stronger antitumor as well as the anti-metastatic effect of the DOX NPs. No significant changes were observed in the spleen, lungs, and kidneys in any of the groups.

**Figure 8.**
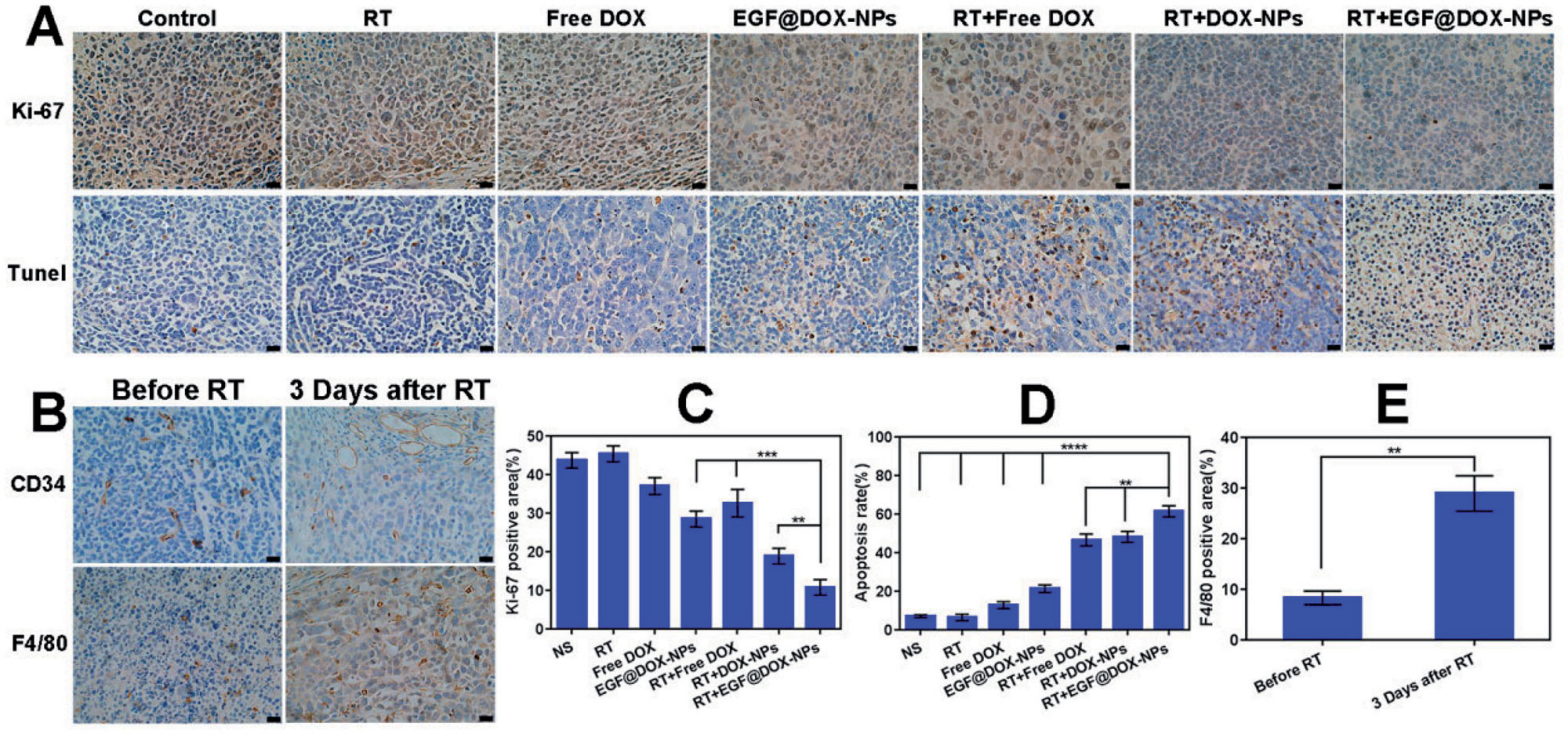
Immunohistochemical analysis. (A) Images of Ki-67 staining and the TUNEL staining. (B) Images of CD34 and F4/80 expression. (C and D) Quantitative analysis of Ki-67 and TUNEL positive areas in different groups. (E) Quantification of F4/80 labeled TAMs in tumor tissues before and after radiotherapy. (Mean ± SD; *n* = 3; Scale bar: 20 µm). ** *p* < .01, *** *p* < .001, **** *p* < .0001.

The percentage of Ki-67+ proliferative cells in the tumor tissues of control and RT-treated mice were 43.71% ± 1.97% and 45.39% ± 2.06%, respectively (*p* > .05). However, treatment with free DOX, EGF@DOX-NPs, RT + free DOX, RT + DOX-NPs, and RT + EGF@DOX-NPs significantly decreased the proliferation of cancer cells. The percentage of Ki-67^+^ cells in these groups was 37.01% ± 2.16%, 28.49% ± 2.06%, 32.59% ± 3.57%, 18.86% ± 2.03%, and 10.77% ± 1.99%, respectively (*p* < .01). TUNEL assay revealed that the apoptosis of cells was highest in the RT + EGF@DOX-NPs group (61.40% ± 2.88%), compared to 7.07% ± 0.7%, 6.37% ± 1,68%, 12.81% ± 1.75%, 21.32% ± 1.99%, 46.57% ± 3.04%, and 48.21% ± 2.83% in the control, RT, free DOX, EGF@DOX-NPs, RT + free DOX, and RT + DOX-NPs groups respectively (*p* < .01) (Figure 8(A,C,D)). These findings further underscore the potent antitumor effects of RT and EGF@DOX-NPs.

The effects of radiation on tumor blood vessels and TAMs were assessed by CD34 and F4/80 immunostaining, respectively. As shown in Figure 8(B,E), RT increased the intratumor vascular diameters and improved the overall morphology after 3 days compared to that in the non-irradiated group. This can improve the blood flow in the tumor tissue and subsequently enhance the accumulation of intravenously injected NPs at the tumor site. Moreover, RT also increased the percentage of TAMs to 28.92% ± 3.49% after 3 days compared to 8.29% ± 1.35% in the non-irradiated group (*p* < .01), suggesting that RT induces infiltration of macrophages into tumor tissues.

### 3.8. *In vivo* micro-PET/CT imaging analysis

**Figure 9.**
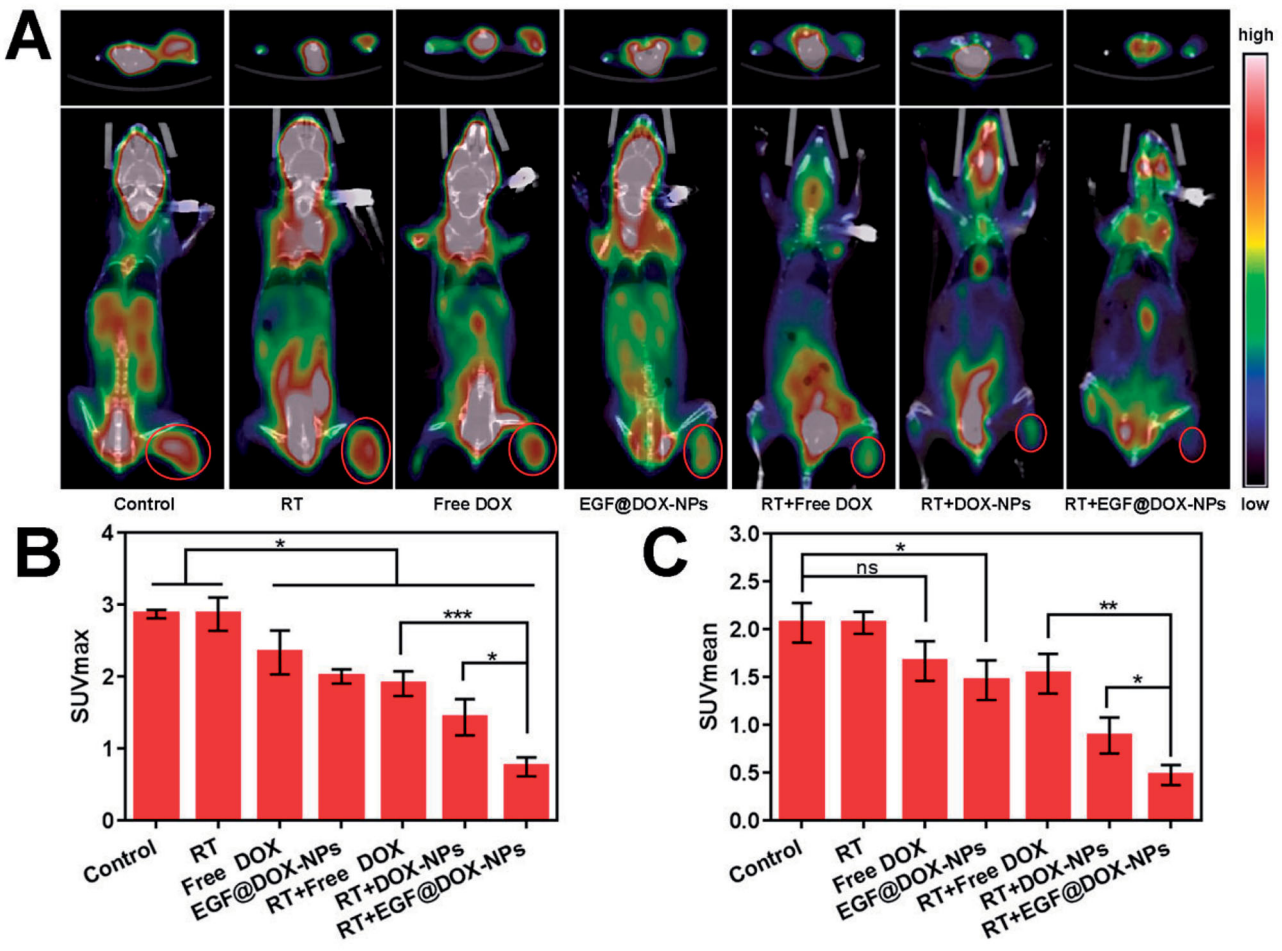
*In vivo* Micro PET/CT scanning. (A) Representative images of tumor tissues of mice treated with different drugs (top: cross section of tumors; bottom: coronal images of mice, red rings indicate the tumor site). (B and C) Maximum value (SUVmax) and the mean value (SUVmean) in tumor tissues. (Mean ± SD; *n* = 3). ns: no significance, * *p* < .05, ** *p* < .01, *** *p* < .001.

Micro-PET-CT imaging of tumor tissues indicated that RT had no effect on glucose metabolism. However, compared to the control and RT groups, RT in combination with EGF@DOX-NPs significantly inhibited glucose metabolism (Figure 9(A)). As shown in Figure 9(B,C), the SUV_max_ and SUV_mean_ of the RT + EGF@DOX-NPs group were lowest among the groups (*p* < .05), which was consistent with the greater antitumor effect of this combination therapy.

### 3.9. Assessment of hematological indicators

The systemic toxic effects of DOX were also evaluated using hematological indices. As shown in Supplementary Figure S4, compared to the control group, the white blood cell (WBC) and platelet (PLT) counts were significantly lower in the free DOX group, but there was no significant difference in the red blood cell (RBC) counts, lymphocyte ratio (LYM-R), hemoglobin (HGB), and plateletocrit (PCT) across the experimental groups. Furthermore, serum alanine aminotransferase (ALT) and aspartate transaminase (AST) levels were high in the DOX, indicative of hepatotoxicity. Also, CK, the cardiac function indicator, was significantly high in the DOX group, whereas DOX-NPs and EGF@DOX-NPs had minimal effect on these parameters. Finally, there was no significant difference in the serum urea, creatinine, and ALB levels across the groups, indicating lack of any renal toxicity. Collectively, encapsulation markedly reduces the side effects of DOX.

## 4. Discussion

The five-year survival rate of highly prevalent lung cancer is very low (Xiong et al., 2016; Li et al., 2021). Radiotherapy and chemotherapy are the primary treatment options for advanced NSCLC. However, the nonselective nature of chemotherapy drugs and damages related to radiotherapy limits their application (Zhang et al., 2019; Liu et al., 2021). Studies show that the therapeutic efficacy of antineoplastic drugs is highly dependent on their selective accumulation in solid tumors, which in turn depends on the pharmacokinetics as well as the TME (Dewhirst & Secomb 2017). The aim of this study was to improve DOX accumulation in tumors using a nanoplatform for targeted delivery and low-dose radiotherapy for modulation of the TME.

NPs were synthesized using the highly biocompatible PELA and PEI polymers (Asadi et al., 2011; Alyafee et al., 2018). The DOX-NPs were coated with EGF through charge neutralization, and the EGF@DOX-NPs actively targeted the A549 cells with high surface EGFR expression and showed lower uptake in the AML-12 and BEAS-2B cells. EGF@DOX-NPs was found to be nontoxic to the liver and kidney. In addition, DOX-induced reduction in the bodyweight of tumor-bearing mice was alleviated by the NPs, indicating that DOX nano-formulations can reduce its side effects, consistent with previous findings (Yang et al., 2017; Zhang et al., 2017). EGFR-medicated endocytosis also enhanced toxicity against tumor cells and consequently stronger inhibition of xenograft growth *in vivo*. EGFR is overexpressed on lung cancer, breast cancer, colorectal cancer, ovarian cancer, and other malignant cells (Li et al., 2012; Shimizu et al., 2019). Several studies have demonstrated the efficacy of treatment of tumors by targeting EGFR. For example, Cheng et al. showed effective selective targeting of tumor tissues using EGF-conjugated NPs (Cheng et al., 2011). Wang et al. also reported a stronger antitumor effect of EGF-modified cisplatin NPs against ovarian cancer (Wang et al., 2013).

Nevertheless, the TME limits the antitumor effect of EGF@DOX-NPs alone. Poor angiogenesis reduces blood flow to tumors, which disrupts the intravenous delivery of chemotherapy agents (Qian et al., 2019). Therefore, improving intratumoral blood flow or local vascular permeability is a feasible strategy for enhancing drug delivery to the tumor site. Radiotherapy can enhance the permeability of blood vessels (Barker et al., 2015; Stapleton et al., 2017), and a single dose of 5 Gy radiotherapy can locally burst the tumor vasculature and promote infiltration of macrophages, significantly increasing drug accumulation in the tumor. In this study, we found that radiotherapy significantly dilated blood vessels in tumor tissues and increased the number of TAMs, conducive for drug delivery to these regions when administered intravenously. Furthermore, the beneficial effect of radiation on drug delivery was directly confirmed by higher DOX concentration at the tumor site in irradiated animals. Potiron et al. showed a significant increase in intratumoral drug concentration post-radiotherapy in two tumor models (Potiron et al., 2019). Also, Kunjachan et al. found that local eruption of tumor blood vessels can enhance the accumulation of drugs at the tumor site (Kunjachan et al., 2015).

In the present study, we designed a novel strategy for the treatment of lung cancer using low-dose radiotherapy to increase vascular permeability and promote the targeted accumulation of drug-loaded NPs, which resulted in a superior antitumor effect through EGFR-mediated endocytosis. Although the impact of radiotherapy increased infiltration of macrophages to the TME, its effect on other immune and stromal cell types was not examined. Therefore, the efficacy of this strategy has to be validated in other solid tumor models. Nevertheless, our study lays the foundation for the combination of radioimmunotherapy and targeted chemotherapy against solid tumors.

## 5. Conclusion

We devised a strategy to enhance tumor ablation by low-dose radiotherapy-induced local vascular outburst that promotes targeted accumulation of drug-loaded NPs at the tumor site. Compared with free DOX, EGF@DOX-NPs exhibited superior cytotoxic against cancer cells both *in vitro* and *in vivo* and was associated with minimal side effects. Accordingly, this strategy is a promising alternative to conventional chemotherapy for treating solid tumors.

## Supplementary Material

Supplemental Material

## Data Availability

The data that support the findings of this study are available from the corresponding author [ShaoZhi Fu: shaozhifu513@163.com] upon reasonable request.
