# Correction

**DOI:** 10.1080/10717544.2024.2350273

**Published:** 2024-05-14

**Article title:** Radiotherapy-induced enrichment of EGF-modified doxorubicin nanoparticles enhances the therapeutic outcome of lung cancer

**Authors:** Jing Wang, Yan Zhang, GuangPeng Zhang, Li Xiang, HaoWen Pang, Kang Xiong, Yun Lu, JianMei Li, Jie Dai, Sheng Lin and ShaoZhi Fu

**Journal:** *Drug Delivery*

**Bibliometrics:** Volume 29, Number 1, pages 588–599

**DOI:** https://doi.org/10.1080/10717544.2022.2036871

When this article was first published online, the following contents and figures were typeset incorrectly.

1. Section 2.1. Synthesis and characterization of PEI-PLA-PEG-PLAPEI copolymer has been corrected as below:
  To synthesize the copolymer PEI-PLA-PEG-PLAPEI (PELI), 1 mM PELA was first dissolved in 40 mL dichloromethane, then 1mM 4-(dimethylamino) pyridine (DMAP) and 3 mM succinic anhydride was added to the PELA solution, the reaction was carried out at room temperature for 24 h under nitrogen atmosphere (N2). The product was precipitated with pre-cooled petroleum ether, vacuum-dried, and 1 mM HOOC-PELA-COOH copolymer, 1mM N-(3-dimethylaminopropyl)-N′-ethylcarbodiimide hydrochloride (EDC), 1 mM N-Hydroxy-succinimide (NHS) was dissolved in 20 mL chloroform for activation 2 h. Finally, 2 mM PEI solution in methanol was slowly added, and the reaction was performed at RT for 24 h under N2. The final product was precipitated in petroleum ether twice, dried in a vacuum oven at room temperature
2. Figures 4 and 5 were incorrect and this been corrected with the below mentioned revised figures.

**Figure 4.**
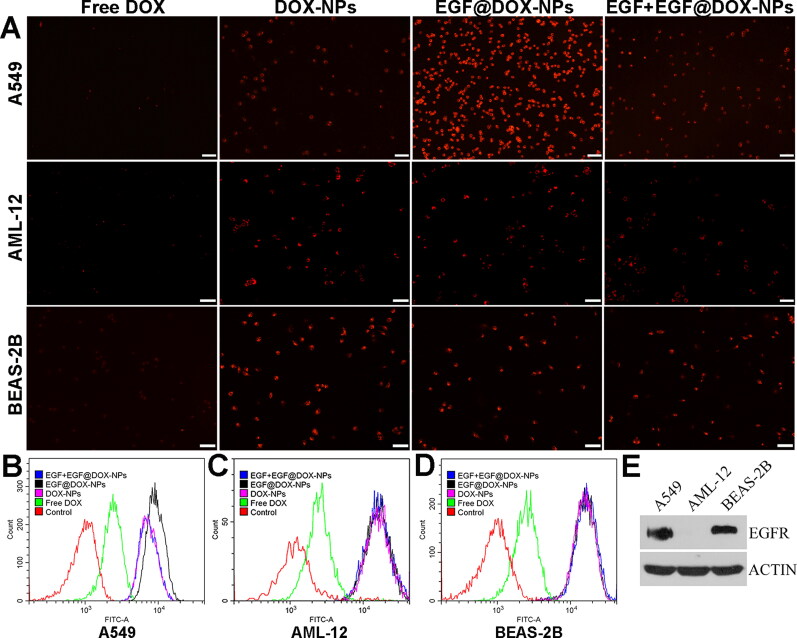
*In vitro* cell uptake. (A) Fluorescence microscopy images showing the uptake of DOX NPs into the EGFR^high^ A549 cells, and the EGFR^low^ AML-12 and BEAS-2B cells. (DOX concentration: 2.5 μg/mL, EGF pretreatment for 4 h in the EGFþEGF@DOX-NPs group; Scale bar: 100 μm). (B–D) DOX content in the A549, AML-12, and BEAS-2B cells was determined by flow cytometry. (E) EGFR protein expression in the A549, AML-12, and BEAS-2B cells was assessed by Western blot analysis. β-Actin expression served as a loading control.

**Figure 5.**
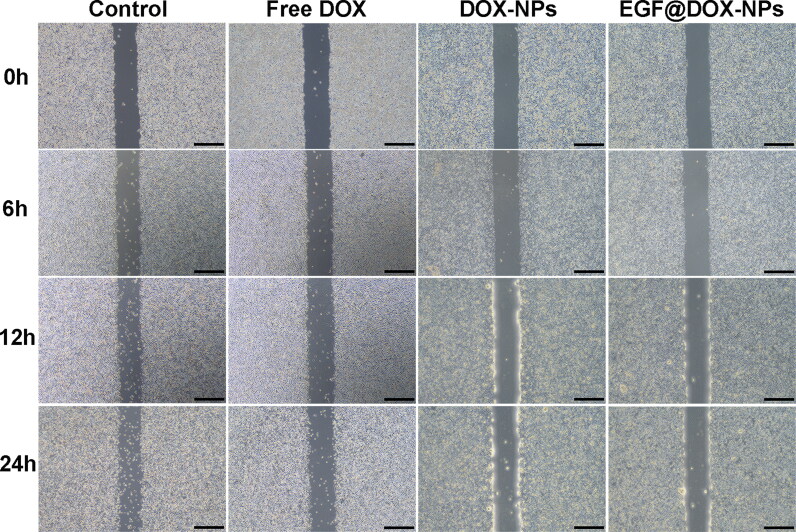
Typical images of wound healing of A549 monolayer after treatment with different drugs for 0, 6, 12, and 24 h (DOX concentration: 1 μg/mL; Scale bar: 500 μm).

Now, the article has been republished online.

