# Supplementary material for: Radiotherapy-induced enrichment of EGF-modified doxorubicin nanoparticles enhances the therapeutic outcome of lung cancer
Source: Drug Deliv. 2022 Feb 14;29(1):588–99. doi: 10.1080/10717544.2022.2036871 (PMC8856057; doi:10.1080/10717544.2022.2036871)
Supplement: Supplemental Material [file IDRD_A_2036871_SM0829.docx]

**Supplementary Materials**

**Radiotherapy Induced Enrichment of EGF-modified Doxorubicin Nanoparticles Enhances the Therapeutic Outcome of Lung Cancer**

Jing Wang^a,#^, Yan Zhang^b,#^, GuangPeng Zhang^a^, Li Xiang^a^, HaoWen Pang^a^, Kang Xiong^a^, Yun Lu^a^, JianMei Li^a^, Jie Dai^a^, Sheng Lin ^a,*^, ShaoZhi Fu^a,*^

*^a^Department of Oncology, the Affiliated Hospital of Southwest Medical University, Luzhou 646000, China*

*^b^Department of Oncology,* *The Affiliated TCM Hospital of Southwest Medical University, Luzhou 646000, China*

Table S1: Preparation of different types of nanoparticles. (Mean ± SD; n = 3).

| Theoretical DL(%) | DOX(mg) | PELI(mg) | Actual DL(%) | Actual EE(%) |
| --- | --- | --- | --- | --- |
| 10%  15% | 10  15 | 90  85 | 9.06±0.09  14.11±0.06 | 90.58±0.87  94.08±0.38 |

**Figure S1**


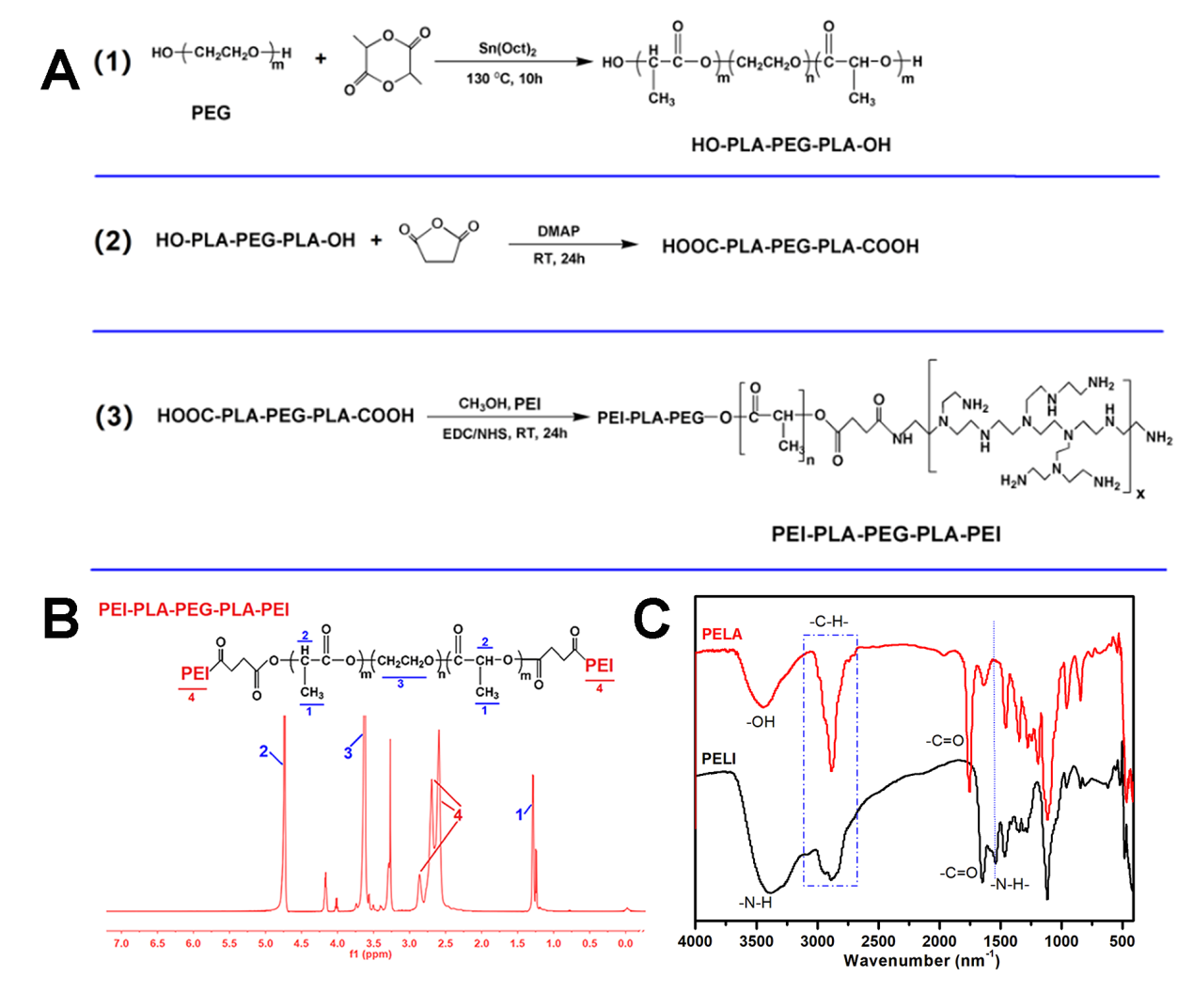


**Figure S1**: Synthesis and characterization of the PELA and PELI copolymers. (A) The synthesis steps of the PELI copolymer. (B) ^1^H-NMR spectrum of the PELI copolymer, (C) FI-TR spectra of the PELA and PELI copolymers.

**Figure S2**


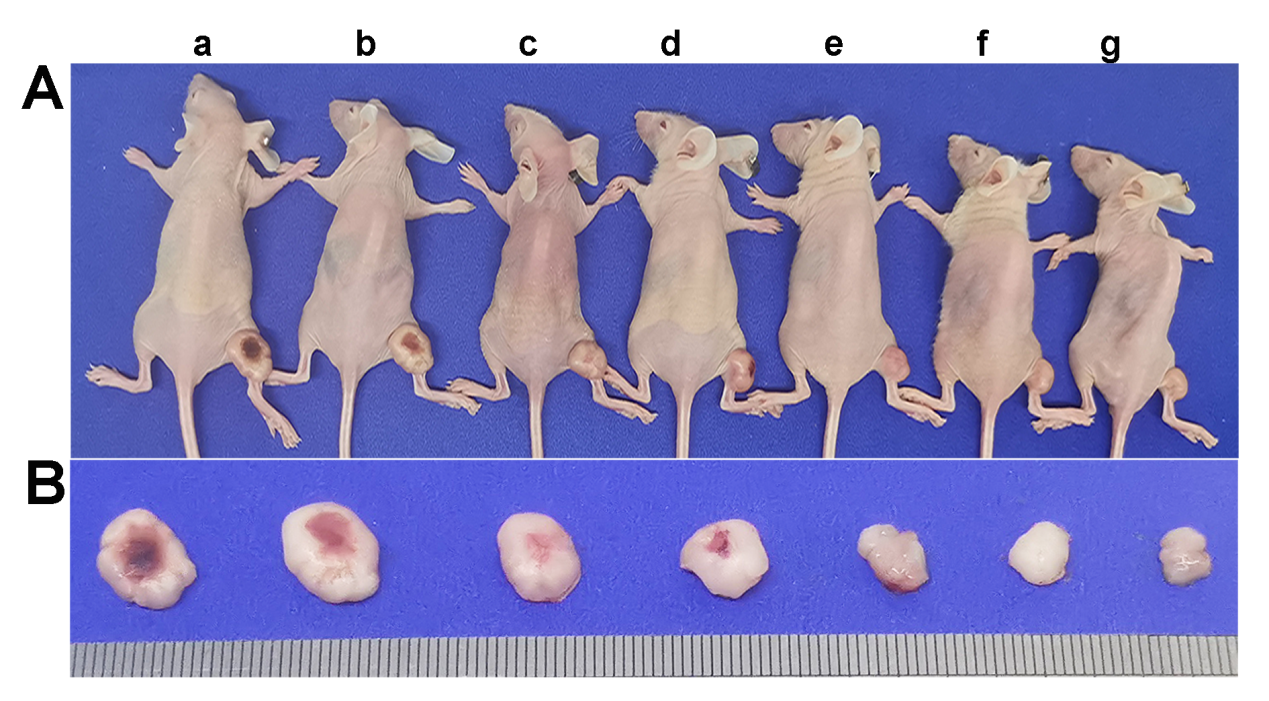


**Figure S2**: In vivo evaluation of antitumor efficacy. (A) Overall appearance photos of mice in different groups. (B) The images of the removed tumors in each group. (a: Control; b: RT; c: Free DOX; d: EGF@DOX-NPs; e: RT+@DOX-NPs; f: RT+EGF@DOX-NPs; g: RT+Free DOX)

**Figure S3**


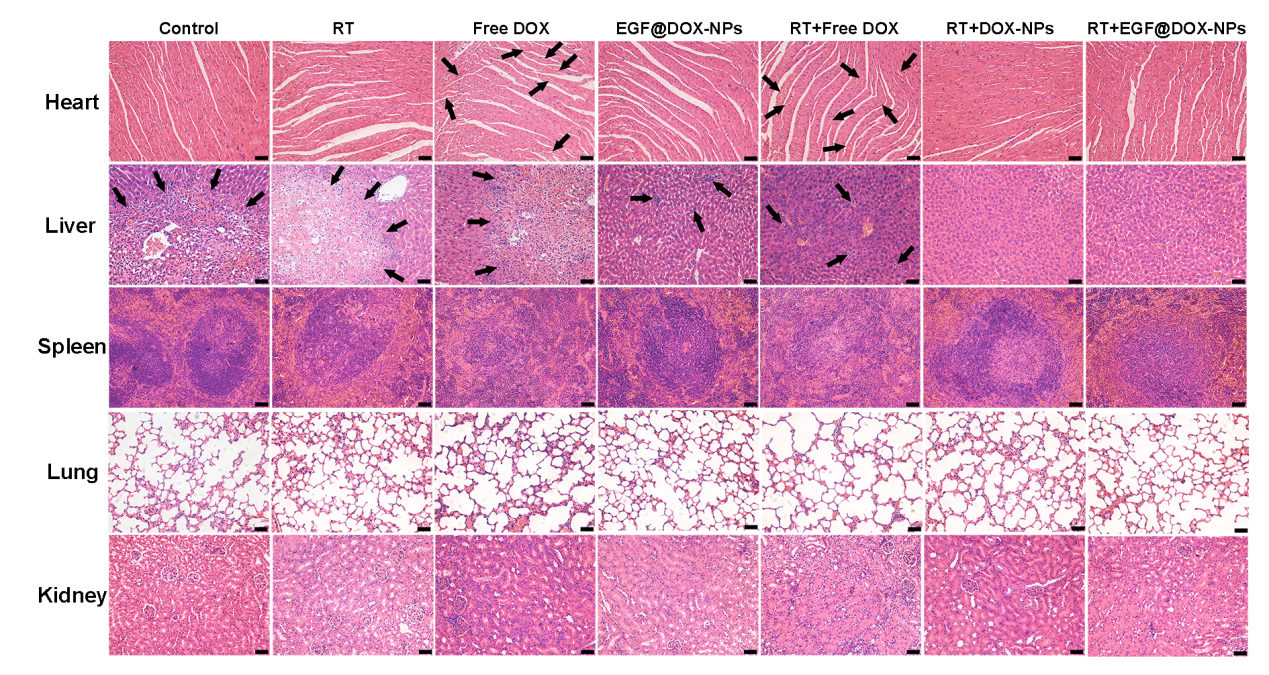


**Figure S3**: Typical images of hematoxylin and eosin (H&E) staining of vital organs (heart, liver, spleen, lung, kidney) in each group. The black arrows in the myocardium shows the myocardium bleeding foci. The black arrows in the liver indicates the metastatic of tumor. (Bar scale: 50µm).

**Figure S4**


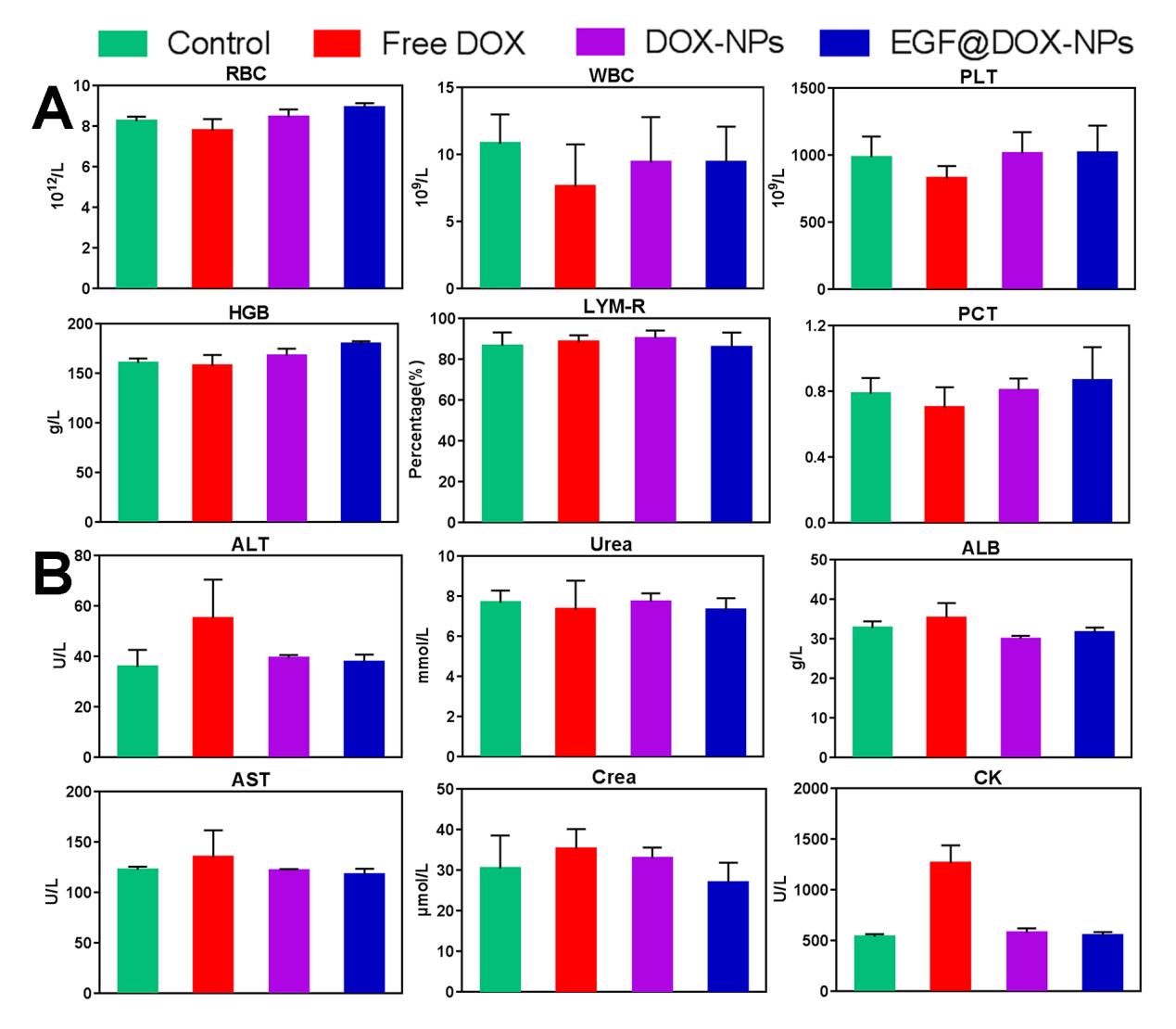


**Figure S4**: *In vivo* evaluation of drug toxicity by analysis of biochemical markers and blood routine indexes. (A) Blood cell analysis included changes in RBC, WBC, PLT, HGB, LYM-R, and PCT after mice treated with free DOX, DOX-NPs, and EGF@DOX-NPs. (B) Changes in liver, kidney, and heart function (including ALT, AST, UREA, CREA, ALB, and CK) after treated with different DOX formulations. (Mean + SD; n=3).
